# Nanoformulation and Evaluation of Oral Berberine-Loaded Liposomes

**DOI:** 10.3390/molecules26092591

**Published:** 2021-04-29

**Authors:** Thuan Thi Duong, Antti Isomäki, Urve Paaver, Ivo Laidmäe, Arvo Tõnisoo, Tran Thi Hai Yen, Karin Kogermann, Ain Raal, Jyrki Heinämäki, Thi-Minh-Hue Pham

**Affiliations:** 1 Faculty of Pharmacy, Duy Tan University, 03 Quang Trung Street, Da Nang 550000, Vietnam; duongthithuan@dtu.edu.vn; 2 Department of Pharmaceutics, Hanoi University of Pharmacy, 13-15 Le Thanh Tong Street, Hoan Kiem District, Hanoi 110403, Vietnam; tranyendhd@gmail.com (T.T.H.Y.); hueptm@hup.edu.vn (T.-M.-H.P.); 3 Institute of Pharmacy, Faculty of Medicine, University of Tartu, 1 Nooruse Street, 50411 Tartu, Estonia; urve.paaver@ut.ee (U.P.); ivo.laidmae@ut.ee (I.L.); karin.kogermann@ut.ee (K.K.); ain.raal@ut.ee (A.R.); 4 Biomedicum Imaging Unit, University of Helsinki, 8 Haartmaninkatu, 00014 Helsinki, Finland; antti.isomaki@helsinki.fi; 5 Institute of Physics, University of Tartu, 1 W. Ostwaldi Street, 50411 Tartu, Estonia; arvo.tonisoo@ut.ee

**Keywords:** berberine, poorly water-soluble alkaloid, liposomes, ethanol-injection, thin-film hydration

## Abstract

Berberine (BBR) is a poorly water-soluble quaternary isoquinoline alkaloid of plant origin with potential uses in the drug therapy of hypercholesterolemia. To tackle the limitations associated with the oral therapeutic use of BBR (such as a first-pass metabolism and poor absorption), BBR-loaded liposomes were fabricated by ethanol-injection and thin-film hydration methods. The size and size distribution, polydispersity index (PDI), solid-state properties, entrapment efficiency (EE) and in vitro drug release of liposomes were investigated. The BBR-loaded liposomes prepared by ethanol-injection and thin-film hydration methods presented an average liposome size ranging from 50 nm to 244 nm and from 111 nm to 449 nm, respectively. The PDI values for the liposomes were less than 0.3, suggesting a narrow size distribution. The EE of liposomes ranged from 56% to 92%. Poorly water-soluble BBR was found to accumulate in the bi-layered phospholipid membrane of the liposomes prepared by the thin-film hydration method. The BBR-loaded liposomes generated by both nanofabrication methods presented extended drug release behavior in vitro. In conclusion, both ethanol-injection and thin-film hydration nanofabrication methods are feasible for generating BBR-loaded oral liposomes with a uniform size, high EE and modified drug release behavior in vitro.

## 1. Introduction

**Figure 1:**
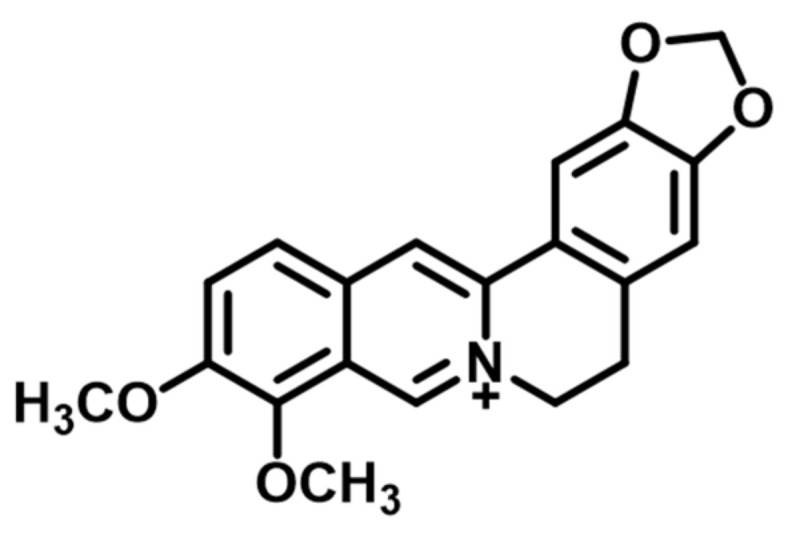
Molecular structure of berberine base form (quaternary isoquinoline).

Berberine (BBR) is a poorly water-soluble quaternary isoquinoline alkaloid (Figure 1) of plant origin. BBR is extracted from the root, rhizome and stem bark of many native plant species including (but not limited to) *Coptis chinensis* Franch., *Coptis japonica* Makino., *Berberis thunbergii* DC., *Hydrastis canadensis* L. and *Thalictrum lucidum* Ait [1]. Untypically to alkaloids, BBR exhibits a yellow color, giving the same characteristic color to plant tissues. BBR possesses biological effects in animal models, such as anti-hypercholesterolemia (lipid peroxidation), anti-inflammatory, anti-diabetic and anti-atherosclerosis activity [2,3,4]. More recently, Allijn et al. (2017) reported that BBR encapsulated in liposomes attenuates cardiac dysfunction after myocardial infarction [5].

The oral route is still the most common and convenient way for the administration of drugs. However, a number of drugs have very low oral bioavailability because of poor water solubility and intestinal permeability, multidrug resistance protein-efflux and metabolic stability [6]. BBR has low oral bioavailability due to a first-pass metabolism effect in the intestinal tract, poor intestinal permeability, self-aggregation, P-glycoprotein mediated efflux and hepatobiliary re-excretion [7,8]. BBR has been shown to induce the activity of multidrug efflux transporter P-glycoprotein (P-gp) in the intestine responsible for active-efflux of drugs from cells, and therefore, less than 10% of oral BBR transports through the intestinal wall [9]. Such limitations associated with the poor oral bioavailability of BBR could be overcome by nanoformulating BBR-loaded liposomes.

Liposomes have found uses in the oral drug delivery of poorly water-soluble drugs. Such drug-loaded liposomes can be fabricated by a wide variety of nanotechnology methods including (but not limited to) ethanol-injection, thin-film hydration, sonication, high-pressure extrusion, reverse-phase evaporation, calcium-induced fusion, dehydration-rehydration, freeze-thaw, microfludization and supercritical fluid methods [10,11]. The key attributes of pharmaceutical liposomes affecting their performance and stability are the liposome size and size distribution, polydispersity index (PDI), morphology, lamellar structure, charge, lipid composition and entrapment efficiency (EE). Liposomes are classified based on their structure into unilamellar vesicles (UV), oligolamellar vesicles (OLV), multilamellar vesicles (MLV) and multivesicular vesicles (MVV). UVs are further classified into small unilamellar vesicles (SUV), medium unilamellar vesicles (MUV), large unilamellar vesicles (LUV) and giant unilamellar vesicles (GUV). Today, a number of powerful analytical methods are available for the characterization of liposomes at a nano-scale [12,13,14]. In our study, we used dynamic light scattering (DLS) (also known as photon correlation spectroscopy, PCS), nanoparticle tracking analysis (NTA), cryogenic electron microscopy (cryo-EM), inverted confocal microscopy and two-photon fluorescence microscopy (TPFM) for the investigation of the size (and size distribution), shape, polydispersity index (PDI), surface morphology, active distribution and internal structure of the BBR-loaded liposomes, respectively.

The liposomes ranging from 100–500 nm in size have the unique ability to enhance the permeability of drugs across the enterocyte barriers and to stabilize drugs [15]. To date, however, little is known about the nanoformulation and applicability of liposomes as nanotechnology drug delivery systems (DDSs) for poorly water-soluble and fairly intestinally permeable BBR of plant origin. Sailor et al. (2015) fabricated BBR-loaded liposomes with a thin-film hydration method and showed that the vesicle size and EE of the liposomes range from 571 nm to 1105 nm and from 56% to 82%, respectively [16]. Nguyen et al. (2014) developed chitosan-coated nano-scale liposomes for the oral delivery of BBR hydrochloride salt [17]. More recently, Luo et al. (2013) introduced novel BBR hydrochloride-loaded long-circulating liposomes fabricated by an ionophore A23187-mediated ZnSO_4_ gradient method [18]. The nanoformulation of DDS (long-circulating liposomes) was optimized yielding the BBR-loaded liposomes with a mean size of 146.9 ± 4.5 nm. Little is known, however, about the distribution and/or migration of poorly water-soluble BBR in the different types of liposomes affecting the loading efficiency, stability and drug release properties of such liposomes.

The aim of this study was to investigate the ethanol-injection and thin-film hydration methods for generating different types of BBR-loaded liposomes and to gain an understanding of the distribution/displacement of BBR in the lamellar structure(s) of such liposomes. Furthermore, the effects of these two nanofabrication methods on the entrapment efficiency (EE) and in vitro drug release of BBR-loaded liposomes were studied. For the size measurements and visualization of liposomes at a nano-scale, we used the complementary powerful nanoparticle tracking analyses (NTA) and microscopy imaging techniques, described in Chapter 4 ‘Materials and methods’ in detail. It is evident that the limitations associated with the poor oral bioavailability of BBR can be overcome using either SUV or MLV-type liposomes as DDSs. The present BBR-loaded liposomes are ultimately intended for reducing non-HDL cholesterol by oral administration.

## 2. Results

### 2.1. Size and Polydispersity Index (PDI) of BBR-Loaded Liposomes

The size and size distribution of the liposomes were determined with two nanoparticle tracking measurement systems, Zetasizer Nano ZSP and ZetaView^®^ (reference is also made to Section 4.4.1 and Section 4.4.2). Table 1 shows the comparison of the size and PDI of the liposomes prepared with both ethanol-injection and thin-film hydration methods. The BBR-loaded liposomes prepared by the ethanol-injection method presented a mean liposome size ranging from 50 nm to 244 nm (Table 1). The BBR-loaded liposomes fabricated by the thin-film hydration method exhibited a large micron-scale size and variation in structure before extrusion. After extruding through a polycarbonate membrane (0.4 μm) 60 times, the mean size of the liposomes reduced significantly and the mean size of the present liposomes ranged from 111 nm to 449 nm (Table 1).

**Figure 2:**
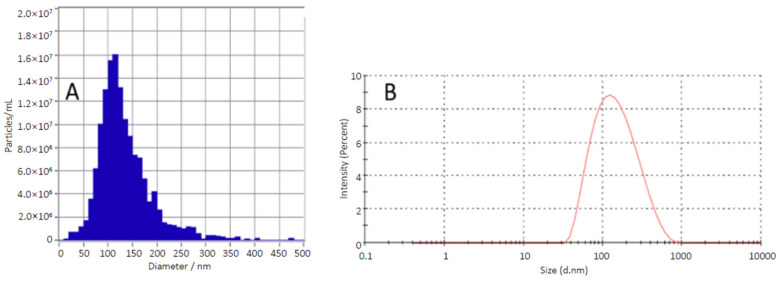
The size and size distribution of berberine (BBR)-loaded liposomes generated by the ethanol-injection method (FJ12): (**A**) The particle size distribution of liposomes (by number of particles per ml) analyzed with ZetaView^®^ NTA; (**B**) The size distribution (by intensity) analyzed with Zetasizer Nano ZSP.

Figure 2 shows the size distribution of BBR-loaded liposomes generated with the ethanol-injection method (FJ12) and analyzed with both abovementioned nanoparticle tracking analysis (NTA) methods (reference is also made to Table 1). ZetaView^®^ NTA was used to measure the size and size distribution (by number of particles per ml) and the scattering movement of liposomes.

### 2.2. Imaging of the Liposomes Generated by the Ethanol-Injection Method

In the Supplementary Material, we show two video clips, Videos S1 and S2, illustrating the scattering movement of BBR-loaded liposomes fabricated by the ethanol-injection method (FJ17 and FJ12). In video clip S.1, the movement of the liposomes (FJ17) can be seen as individual, separate light spots, suggesting that the great majority of the liposomes is not agglomerated. However, some clusters of light spots can be observed, indicating that the present liposomes (FJ12) are partially agglomerated (Supplementary Material Video S2).

**Figure 3:**
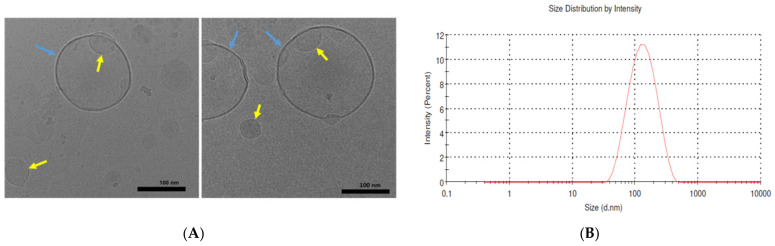
(**A**) Cryogenic electron microscopy (Cryo-EM) images of the BBR-loaded liposomes fabricated by the ethanol-injection method (FJ21). The micrographs show the unilamellar vesicle (UV) structure of the liposomes. Scale bar = 100 nm. (**B**) The size distribution of the corresponding liposomes (FJ21) determined by the Zetasizer Nano ZSP system.

The morphology and structure of BBR-loaded liposomes fabricated by the ethanol-injection method were also imaged by means of cryogenic electron microscopy, Cryo-EM (Figure 3). Cryo-EM enabled to image the size and morphology of small-size liposomes obtained with an ethanol-injection method. As shown in Figure 3A, the liposomes (FJ21) were spherical and unilamellar vesicles (UVs) with a single, thin layer surrounding the entrapped aqueous phase. This thin layer is composed of phospholipids and BBR. In addition to the liposomes over 100 nm in diameter (blue arrows), a number of smaller liposomes with a size of ca. 50 nm are visible (yellow arrows). The mean size of the liposomes (FJ21) determined by the Zetasizer Nano ZSP system was 117.3 ± 1.3 nm (Figure 3B, Section 4.2). The size distribution of the liposomes, however, was quite broad, ranging from 38 nm to 459 nm (Figure 3B).

**Figure 4:**
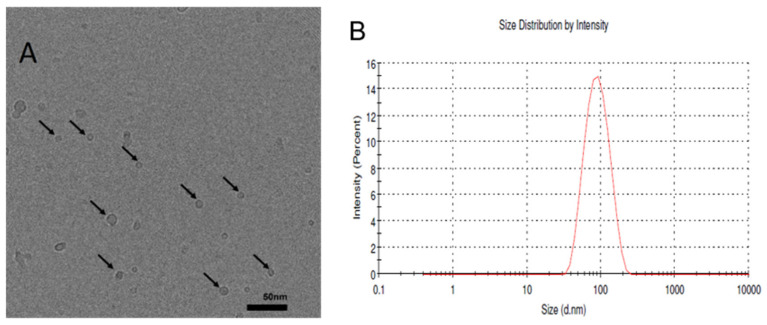
(**A**) The cryogenic electron microscopy (Cryo-EM) image showing the formation of BBR-loaded small liposomes (mixed micelles) (FJ17) with sodium deoxycholate (SDC) in the composition. The liposomes were generated by the ethanol-injection method. Scale bar = 50 nm. (**B**) The size distribution of the corresponding liposomes (FJ17) determined by the Zetasizer Nano ZSP system.

Figure 4 shows the Cryo-EM image on the formation of BBR-loaded small liposomes (mixed micelles) (FJ17) composed of sodium deoxycholate (SDC) and generated by the ethanol-injection method (Figure 4A). Figure 4B shows the size distribution of the corresponding liposomes determined by a Dynamic Light Scattering (DLS) measuring system (Zetasizer Nano ZSP). The liposomes measured by DLS ranged from 38 nm to 220 nm.

### 2.3. Imaging of the Liposomes Generated by the Thin-Film Hydration Method

BBR, as an autofluorescent drug, enables the use of confocal fluorescence microscopy for imaging the internal structure and drug distribution of BBR-loaded liposomes. We found that the application of fluorescence microscopy for imaging the liposomes generated by an ethanol-injection method was extremely challenging due to their small size. Therefore, we were able to use fluorescence microscopy only for imaging of the larger-size liposomes generated by a thin-film hydration method. Fluorescence microscopy enabled to visualize (map) the distribution of autofluorescent BBR in the liposomes.

**Figure 5:**
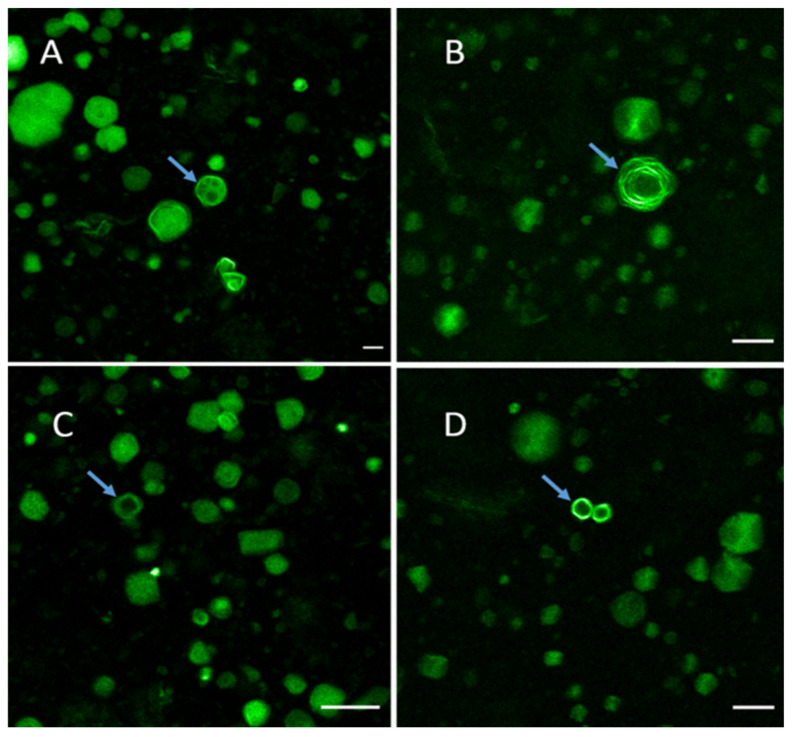
Fluorescence micrographs revealing the various types and internal structures of the BBR-loaded liposomes (before extrusion) generated by the thin-film hydration method (FH12): (**A**) Multivesicular vesicles (MVV); (**B**) Multilamellar vesicles (MLV); and (**C,D**) unilamellar vesicles (UV). Scale bars = 10 μm.

**Figure 6:**
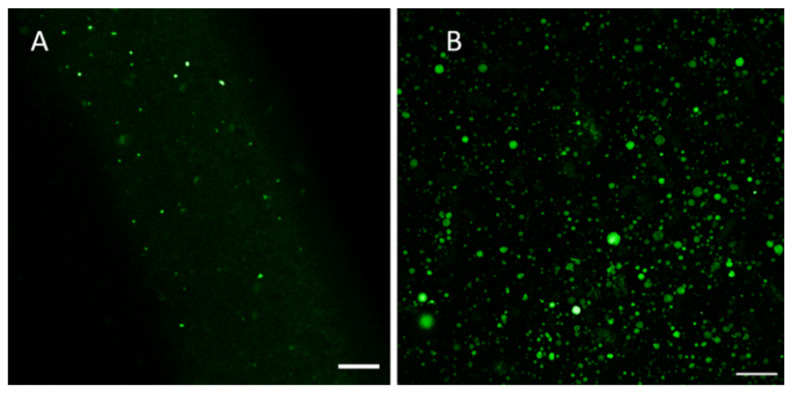
Fluorescence micrographs revealing the BBR-loaded liposomes (before extrusion) generated by the thin-film hydration method: (**A**) Sample FH21, scale bar 10 μm, wavelength 802.9 nm; (**B**) Sample FH12, scale bar 50 μm, wavelength 488 nm.

Figure 5 and Figure 6 show some representative fluorescence micrographs (with a high magnification) on the internal structure of the liposomes generated by the thin-film hydration method (FH12) prior to extrusion through a polycarbonate membrane with a pore size of 0.4 μm. The present liposomes as ‘thin-film hydration intermediate products’ presented a wide variation in size, shape and internal structure. The mean size of the liposomes (FH12) prior to extrusion ranged from 800 nm to 1000 nm (Figure 5). The liposomes were spherical in shape and presented no aggregation or cluster formation. BBR was entrapped and distributed inside the liposomes. Figure 6 shows the fluorescence micrographs on the more representative samples of BBR-loaded liposomes (before extrusion) generated by the thin-film hydration method (FH12, FH21). Unfortunately, we were not able to visualize the internal structure of final nano-size liposomes (after extrusion) and the liposomes generated by the ethanol-injection method due to the limited resolution of a confocal fluorescence microscope at a nano-scale.

**Figure 7:**
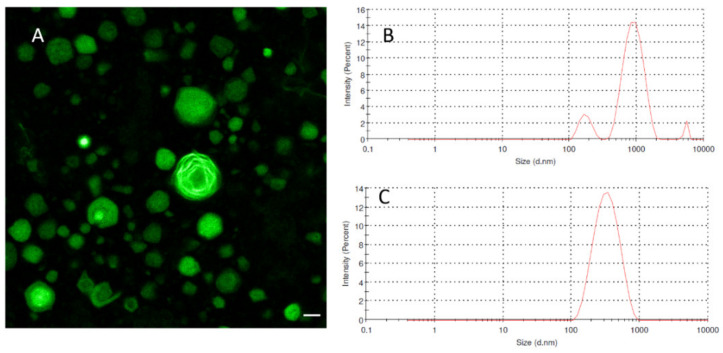
(**A**) Fluorescence micrograph showing the BBR-loaded ‘intermediate’ liposomes (before extrusion), scale bar = 10 μm; (**B**) Zetasizer Nano ZSP graph showing the size distribution of the BBR-loaded liposomes before extrusion; (**C**) Zetasizer Nano ZSP graph showing the size distribution of the final liposomes (after extrusion) generated by the thin-film hydration method (FH12).

Figure 7 presents the representative confocal fluorescence microscopy image (with a high magnification) of the BBR-loaded liposomes (FH12) generated by the thin-film hydration method (before extrusion) and the size distribution of “intermediate” liposomes (before extrusion) and final nano-scale liposomes measured by Zetasizer Nano ZSP. As seen in Figure 7A, autofluorescent BBR was successfully entrapped and distributed (layered) inside the liposomes. The corresponding final drug-loaded liposomes (after extrusion) exhibited a very uniform nano-scale size as shown in the size distribution of liposomes (FH12) determined by Zetasizer Nano ZSP (Figure 7C).

### 2.4. Entrapment Efficiency (EE) of Liposomes

**Figure 8:**
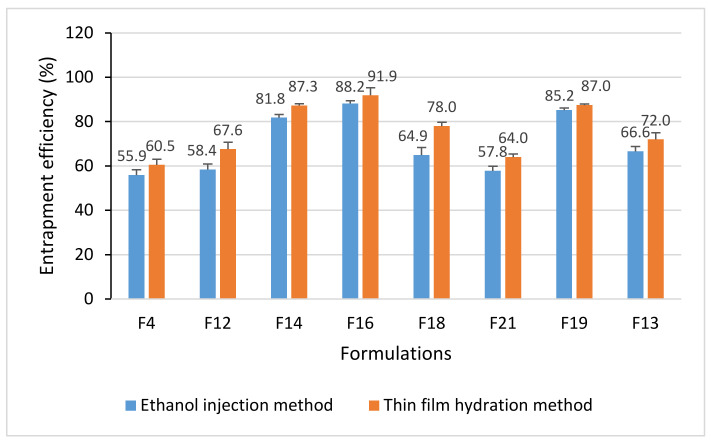
Entrapment efficiency (EE) of the berberine (BBR)-loaded liposomes generated by ethanol-injection (FJ) and thin-film hydration (FH) methods (*n* = 3, mean ± SD). Error bars show the standard deviation between three repeat measurements.

Figure 8 shows the EE of BBR-loaded liposomes fabricated in both nanofabrication methods (reference is also made to Section 4.4.3). With the thin-film hydration method, the EE of liposomes ranged from 60.5% to 91.9%, and with the ethanol-injection method from 55.9% to 88.2% (Figure 8). The difference in EE between the nanofabrication methods was statistically significant (*p* < 0.05). We also investigated the effects of a molar ratio of lipids (HSPC:DSPG) on the EE of liposomes (Figure 8; the formulation codes are shown in Table 1). The EE of the BBR-loaded liposomes increased in the following order (varying the HSPC:DSPG molar ratio within the formulations): FJ18 (HSPC:DSPG 7:3) 64.9%, FH18 (7:3) 78.0%, FJ14 (6:4) 81.8%, FH14 (6:4) 87.3%, FJ16 (4:6) 88.2%, FH16 (4:6) 91.9%, FJ13 (6:4) 66.6%, FH13 (6:4) 72%, FJ19 (4:6) 85.2%, FH19(4:6) 87.0%.

### 2.5. Drug Release In Vitro

**Figure 9:**
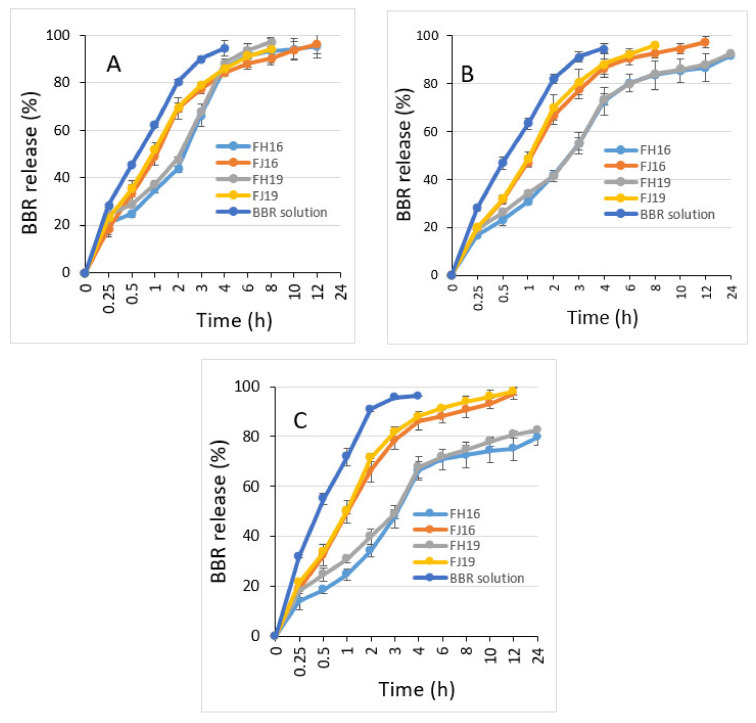
The in vitro berberine (BBR) release of the liposomes prepared by the thin-film hydration (FH16, FH19) and ethanol-injection (FJ16, FJ19) methods. (**A**) The dissolution tests (*n* = 3) were carried out in three different dissolution media (at 37 °C). (**B**) Diluted 0.1 N hydrochloric acid (HCl) solution (pH 1.2). (**C**) Phosphate buffer saline (pH 4.5). Phosphate buffer saline (pH 6.8).

Figure 9A–C shows the in vitro drug release behavior of BBR-loaded liposomes fabricated by the ethanol-injection (FJ16, FJ19) and thin-film hydration (FH16, FH19) methods (reference is also made to Section 4.4.4). The liposome formulations FJ16 and FH16 were selected for the in vitro drug release tests, since they showed the highest EE of BBR among the formulations studied. The present liposome formulations with a high EE will be also used in the further in vivo studies with an animal model. The in vitro drug release studies were conducted also with the FJ19 and FH19 liposome formulations, which showed the high EE of BBR and a high BBR-lipid ratio.

The release rate of BBR from the liposomes fabricated by ethanol injection was higher than that observed with the liposomes generated by thin-film hydration in all three dissolution media studied. As seen in Figure 9A, the total amount of BBR released from the liposomes in a gastric-simulating 0.1 N HCl solution (pH 1.2) was more than 80% within the first 4 h. The liposomes prepared by the thin-film hydration method (FH16, FH19) released BBR in an acidic medium (pH 1.2) slower than the liposomes prepared by the ethanol-injection method (FJ16, FJ19).

The BBR release of liposomes (FJ16, FH16, FJ19 and FH19) was slightly different in the phosphate buffer saline dissolution medium (pH 4.5) compared with that observed in a 0.1 N HCl medium (pH 1.2). Within 8 h, the total amount of BBR released from the FH16 and FH19-type liposomes was approximately 80%, while the amount of BBR released from the FJ16 and FJ19-type liposomes within 8 h was over 90% (Figure 9B). The difference in the release rate of BBR from the liposomes fabricated by thin-film hydration (FH16, FH19) was even more evident in a phosphate buffer saline medium (pH 6.8) (Figure 9C). The FH16 and FH19-type liposomes presented a BBR release of approximately 75% within 8 h. The corresponding percentage in drug release for FJ16 and FJ19-type liposomes was over 90%.

### 2.6. Physical Solid-State Properties of Liposomes

**Figure 10:**
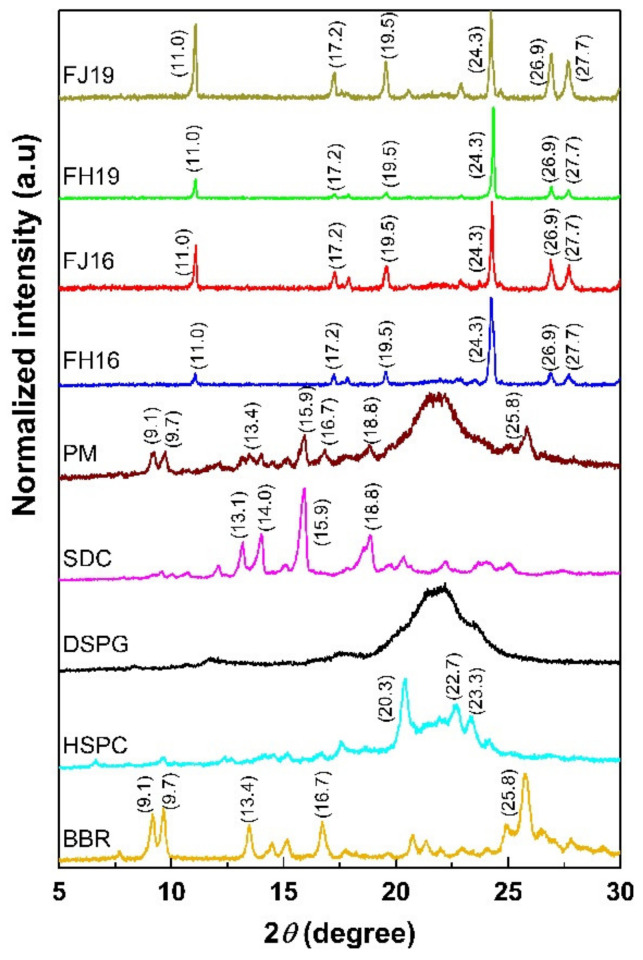
XRPD patterns of berberine (BBR) in powder form, distearoyl phosphatidylglycerol (DSPG), hydrogenated soy phosphatidyl choline (HSPC), physical mixture (PM) of BBR, HSPC, DSPG and SDC and BBR-loaded liposomes generated by the ethanol-injection (FJ16, FJ19) and thin-film hydration methods (FH16, FH19).

Since the ethanol-injection method produced smaller and more uniform size liposomes than the thin-film hydration method, we investigated the solid-state properties of these FJ and FH liposomes in more detail. Figure 10 shows the XRPD patterns of the materials in powder form (BBR, DSPG, HSPC, SDC), the physical mixtures (PM) of BBR, HSPC, DSPG and SDC and the dried powder of liposomes fabricated by the ethanol-injection method (FJ16, FJ19) and the thin film hydration method (FH16, FH19). BBR in powder form is a crystalline solid exhibiting multiple sharp and characteristic diffraction peaks at 9.1°, 9.7°, 13.4°, 16.7° and 25.8° 2*θ* (Figure 10). DSPG presented one wide reflection between 19.6° and 24.2° 2*θ* (Figure 10). HSPC showed characteristic peak reflections at 20.3°, 22.7° and 23.3° 2*θ*. SDC revealed as a crystalline form with shape peaks at 13.1°, 14°, 15.9° and 18.8° 2*θ*.

The XRPD trace of PM presented the main characteristic reflections of all liposome components, in particular the characteristic peaks for BBR at 9.1°, 9.7°, 13.4°, 16.7° and 25.8° 2*θ* (Figure 10). In contrast, the sharp characteristic peaks for BBR at 9.1°, 9.7°, 13.4°, 16.7° and 25.8° 2*θ* disappeared in the XRPD pattern of the BBR-loaded liposomes. Instead, a number of other peaks appeared at 11°, 17.2°, 19.5°, 24.3°, 26.9° and 27.7° 2*θ*.

## 3. Discussion

We investigated the effects of both composition and method of preparation on the formation and final properties of BBR-loaded liposomes. Our results showed that the BBR-loaded liposomes prepared by the ethanol-injection method are smaller in size and have a more uniform size distribution than the corresponding liposomes prepared by the thin-film hydration method (Table 1). These differences are obviously due to the different formation mechanisms of liposomes involved in these two fabrication methods. In an ethanol-injection method, liposomes are formed from BBR, lipids and the other excipients, when an organic solvent phase (ethanol) is removed from an aqueous phase under vacuum. In a thin-film hydration method, BBR and the excipients are first dissolved in the binary mixture of organic solvents, then the solvent system is evaporated under vacuum, and the liposomes are formed from a dried thin-film layer after hydration by distilled water. The size of the liposomes generated by a thin-film hydration method needs to be further reduced with an ultrasonication extrusion or related methods [19].

The results obtained with nanoparticle tracking analysis (ZetaView^®^ NTA and Zetasizer Nano ZSP) verified that the size and size distribution of the present BBR-loaded liposomes are in a nano-scale range and are uniform. However, we also found that the HSPC:DSPG molar ratio affects the formation or fusion of a phospholipid bilayer in the liposomes, and thus, could have an impact on the final size of BBR-loaded liposomes (Table 1). The advantage of preparing liposomes with the ethanol-injection method is that liposomes are not exposed to external treatments such as extrusion or sonication. These treatments have been reported to induce a risk of drug leakage of the liposomes in single bilayer liposomes and large-volume liposomes [20,21]. The PDI values for the liposomes generated with both nanofabrication methods were smaller than 0.3, suggesting the narrow size distribution of the liposomes (Table 1).

The Cryo-EM results show that the BBR-loaded liposomes fabricated by the ethanol-injection method consist of both small unilamellar vesicles (SUVs) and large unilamellar vesicles (LUVs). The largest BBR-loaded liposomes (FJ21) were several hundred nanometers in diameter. We also found that the formation and final structure of BBR-loaded liposomes are highly influenced by the liposome formulation. Sodium deoxycholate (SDC) used in the present liposomes is known as an agent for increasing the oral bioavailability of drugs [22,23,24] and enhancing the stability of liposomes in the intestinal tract [25]. In our study, SDC was added to the liposomes (FJ17) generated by the ethanol-injection method, and the present liposomes were compared with those (FJ21) obtained without SDC and with a different molar ratio of α-TP (Table 1). Since α-TP is a phospholipid-bilayer stabilizer, it enables the reduction of the leakage of contents from vesicles [26,27].

We found that the average size of BBR-loaded liposomes containing SDC (FJ17) was much smaller than that of liposomes without SDC (FJ21) (Table 1, Figure 3; Figure 4). The SDC-induced formation of small liposomes or mixed micelles (FJ17) ranging from 5 nm to 10 nm were also observed in the Cryo-EM image (Figure 4A). This finding is in accordance with the results of Zheng et al. (2012), who reported the corresponding effects of SDC on the formation of itraconazole transferosomes [28]. We also found that nano-liposomes or mixed micelles with a diameter less than 10 nm can be imaged by means of Cryo-EM but imaging such liposomes with a DLS method (Zetasizer Nano ZSP) is much more challenging.

Perhaps surprisingly, the size of the liposomes (40–220 nm) measured by DLS differs from the liposome size obtained with Cryo-EM imaging (Figure 4). Our results, however, are in line with the results of Crawford et al., who also reported differences in liposome sizes measured by Cryo-EM and DLS. Cryo-EM was found to detect even the smallest liposomes having a diameter of 20 nm (a low-PEG/high-cholesterol liposome formulation), which the DLS method was not able to detect [29]. The differences in the measurement systems (Cryo-EM, DLS) could be explained by the bias of DLS in detecting small nano-scale lipid particles in a poly-dispersed suspension [30].

The PDI value for the BBR-loaded liposomes generated by the ethanol-injection method (FJ17) was 0.113, suggesting that the size distribution of liposomes is not monomodal. The mean size of liposomes measured by the Cryo-EM and DLS techniques are shown to be similar only if the size distribution is monomodal and monodisperse [29]. Furthermore, there are many factors affecting the size and PDI results of liposomes obtained with DLS, such as temperature [31], concentration of particles [32], pH and viscosity of the buffer [33]. Cryo-EM has been reported to be a golden standard for liposome imaging, enabling the simultaneous size measurement and imaging of the morphology and sub-structure of liposomes [34,35]. The application of Cryo-EM, however, is still limited in the analysis of pharmaceutical liposomes due to the relatively complex sample preparation, cost and perhaps also challenges in the number of liposomes to be trapped in EM grids [29].

We found that the thin-film hydration method generates a number of different types of liposomes (such as MVV, MLV and UV) prior to their extrusion through a polycarbonate membrane with a pore size of 0.4 μm (Figure 5). The mean diameter of these BBR-loaded liposomes as ‘thin-film hydration intermediate products’ was in the range of 800 nm and 1000 nm. The present results on liposome formation and liposomal structures in thin-film hydration are in good agreement with the results of Kim et al. [36] and Uhl et al. [37]. In our study, the size of the MVV-, MLV- and UV-type liposomes ranged from some hundred nanometers to a few micrometers (up to approximately 10–13 μm). The final BBR-loaded liposomes generated by the thin-film hydration method (after extrusion), however, presented a symmetrical nano-scale size and a very homogenous size distribution (Figure 7).

Auto-fluorescent BBR was found to be successfully entrapped inside the liposomes and to accumulate in the bi-layered phospholipid membrane of the liposomes (Figure 5). This finding is perhaps surprising, since poorly water-soluble BBR is a hydrophilic compound [38]. The solubility of BBR in water and phosphate buffer pH 7.0 at 37 °C is 8.50 ± 0.40 mM and 9.69 ± 0.37 mM, respectively [38]. Our finding differs from the results of earlier studies, where BBR was reported to be distributed in the aqueous core of the liposomes generated by the film hydration or ethanol-injection method [5,39]. According to the literature, there are two ways to load drugs into liposomes: one is passive loading (i.e., the drug is encapsulated during the formation of liposomes) and the other is active loading (i.e., the drug is entrapped after the generation of liposomes) [40]. In our study, BBR was loaded into the liposomes by the passive loading method, significantly differing from the active method applied in the abovementioned studies.

The EE for BBR-loaded liposomes studied here ranged from 56% to 92% (Figure 8). It is evident that these differences in EE are due to the differences in the structure of liposomes. The BBR-loaded liposomes obtained with the thin-film hydration method were UV, MLV or MVV in structure, while the structure of liposomes obtained with the ethanol-injection method was only UV. Nguyen et al. reported that the EE of the BBR hydrochloride-loaded liposomes generated with the thin-film hydration method was 83.2 ± 0.4%, which is in line with the EE results obtained in our study [17]. Luo et al. reported that the EE of the liposomes loaded with BBR hydrochloride and prepared with a modified ethanol-injection method was 94.3 ± 2.1%. They also used a constructed transmembrane ionophore A23187-mediated ZnSO_4_ gradient in the ethanol-injection method [18]. The drug-lipid ratio, however, was 1:20 (*w/w*) in the study reported by Luo and co-workers (2013), while the lowest molar drug-lipid ratio in our study was 6:9 (Table 1).

We also found that the molar ratio of lipids (HSPC:DSPG) used in the nanoformulation significantly affects the EE of BBR-loaded liposomes generated by both fabrication methods studied (Figure 8). This observation could be explained by the charge difference between distearoyl phosphatidyl glycerol, DSPG (a negative charged molecule) and BBR (a positive charged molecule), resulting in the tighter binding of BBR on the bilayer phospholipid membrane of the liposomes as the DSPG molar ratio is increased in the liposome formulations. DSPG is used as a (bi-)layer-forming material in liposome formulations.

As shown in Figure 9, all BBR-loaded liposomes studied presented extended drug release behavior in vitro. The release rate of BBR from the liposomes fabricated by the ethanol-injection method was higher than that observed with the liposomes generated by thin-film hydration in all three dissolution media studied. With virtually all liposome formulations studied (excluding FH16 and FH19 at pH 6.8), the amount of BBR released within 8 h was over 75% in all dissolution media tested. The liposomes prepared by the ethanol-injection method released BBR in a gastric-simulating 0.1 N HCl solution (pH 1.2) slightly faster within the first 4 h than the liposomes prepared by the thin-film hydration method. These results are in accordance with the recent studies reporting that liposomes are chemically unstable in gastric acid, thus resulting in differences in their dissolution properties [41].

In phosphate buffer saline medium (pH 4.5 and pH 6.8), the total amount of BBR released from the liposomes generated by the thin-film hydration method (FH16, FH19) was approximately 75–85% within 8 h, while the amount of BBR released from the liposomes generated by the ethanol-injection method (FJ16, FJ19) was over 90%. The difference in the release rate of BBR could be explained by the differences in the internal structure of the liposomes generated by these two fabrication methods (as seen in Figure 3, Figure 4 and Figure 5). It is evident that due to the unilamellar structure, the FJ16 and FJ19-type liposomes are disrupted faster by gastric acid compared with the FH16-type liposomes with a multiple-layer structure. The BBR release of F19-based liposomes was slightly faster than that observed with the F16-based liposomes. This could be explained by the higher molar ratio of BBR in the liposome formulation F19 compared with the formulation F16. It is evident that the higher molar ratio of BBR in the liposomes makes the lipid bilayers as less dense. This is obviously due to the accumulation of BBR in the phospholipid bilayers as shown in Figure 5 and Figure 7. The slower BBR release of liposomes fabricated by thin-film hydration (FH16 and FH19) in pH 6.8 is in good agreement with the corresponding results reported in the literature [16]. The present extended-release drug release behavior of BBR from liposomes could be a promising strategy to improve the oral bioavailability of BBR in the gastrointestinal tract.

Since the ethanol-injection method produced more uniform-sized and structured liposomes than the thin-film hydration method, we investigated the solid-state properties of BBR-loaded liposomes in more detail (Figure 10). We found that a number of sharp characteristic peaks for BBR at 9.1°, 9.7°, 13.4°, 16.7° and 25.8° 2*θ* disappeared in the XRPD pattern of the BBR-loaded liposomes. Instead of those peaks, some new peaks appeared at 11°, 17.2°, 19.5°, 24.3°, 26.9° and 27.7° 2*θ*. The XRPD patterns could be explained by the transition of the characteristic peaks of BBR or other components. This means that the spacing between the crystal lattice planes (d) of the atoms changes because of the change of an angle 2*θ* according to Bragg’s Law, Equation (1):

(1) $$d=\frac{n\lambda }{2\sin\theta }$$

where *n* (an integer) is the ‘order’ of the reflection, λ is the wavelength of incident X-rays, d is the interplanar spacing of the crystal and *θ* is the angle of incidence (*θ* = 2*θ*/2).

Our results suggest that the BBR loaded in the liposomes has partially lost its crystallinity, or alternatively, BBR is associated more firmly with the other components used in the liposomes. These findings are in line with the results of Xue et al. (2013), who reported that the solid state of BBR changed from a crystal form to an amorphous form when dispersed in lipid nanoparticles [42]. As shown in Figure 10, the intensity of all peaks in the XRPD patterns of FH16 and FH19-type liposomes was weaker than the corresponding peak intensities observed with the FJ16 and FJ19-type liposomes. This suggests that the solid-state change of BBR in the FH16 and FH19-type liposomes was more prominent than that in the FJ16 and FJ19-type liposomes. Alternatively, BBR could be also merged with other components of the liposomes, especially in FH16-type liposomes.

## 4. Materials and Methods

### 4.1. Materials

Berberine (Mw 336.36 g/mol and chemical purity >98%; Sichuan Weikeqi Biological Technology Co., Ltd., Chengdu, China) was used as an active ingredient in base form (C_20_H_18_NO_4_^+^). Hydrogenated soy phosphatidyl choline (HSPC) (>98%) and distearoyl phosphatidyl glycerol (DSPG) (>98%) were obtained from Lipoid GmbH, Ludwigshafen, Germany. Alpha-tocopherol (α-TP) was purchased from CISME Italy s.r.l., Milano, Italy and sodium deoxycholate (SDC) (>98%) was purchased from Bomeibio Co. Ltd., Hefei, China. Absolute ethanol (>99.8%) (Honeywell, Riedel-de Haën, Seelze, Germany) and methanol and chloroform (purchased from Lach-ner, s.r.o., Brno, Czech Republic) were used as solvents for the fabrication of liposomes.

### 4.2. Preparation of Liposomes by the Ethanol-Injection Method

The compositions and final product characteristics of all experimental liposome formulations prepared by the ethanol-injection and thin-film hydration methods are summarized in Table 2. For fabricating liposomes, BBR, HSPC, DSPG, SDC and α-TP were used in different molar ratios in both the ethanol-injection and thin-film hydration methods. In the ethanol-injection method [20], BBR, HSPC, DSPG, SDC and α-TP were dissolved in absolute ethanol. The solution was then injected in distilled water at 65 °C while stirred at 500 rpm. The injection rate was 1.0 mL/min. Ethanol was evaporated at 60–65 °C for 30–60 min under vacuum by a rotary evaporator (R-300, Büchi Labortechnik GmbH, Essen, Germany) at 150 rpm to form the suspension of liposomes. The suspensions were stored at 2–8 °C prior to characterization.

### 4.3. Preparation of Liposomes by Thin-Film Hydration

For preparing the liposomes, we used the thin-film hydration method described by Bangham et al. [43] with slight modifications. BBR, HSPC, DSPG, SDC and α-tocopherol were dissolved in the mixture of methanol and chloroform at a volume ratio of 2.5:1. This solution was evaporated at 50–55 °C under vacuum (300–350 mPa) to form a thin-film layer on the wall of a flask in a rotary evaporator. The rotation rate of an evaporation flask was 150 rpm. The rotary evaporation was continued until all trace of organic solvents in the wall of a flask was removed to ensure the absence of the solvents. The dried thin-film layer was then hydrated completely by distilled water to form the suspension of liposomes. Finally, the size of the liposomes was reduced by extruding through a polycarbonate membrane with a pore size of 0.4 μm using a mini-extruder (Avanti Polar Lipids, Inc., Alabaster, AL, USA) equipped with the Hamilton syringe (The Hamilton Company, Boston, MA, USA). The suspensions were stored at 2–8 °C prior to characterization.

### 4.4. Characterisation of Liposomes

#### 4.4.1. Size and Polydispersity Index (PDI)

The aqueous suspension of liposomes was diluted 100-fold with distilled water, and subsequently, the size and PDI were determined using a DLS measuring system Zetasizer Nano ZSP (Model ZEN 5600, Malvern Panalytical Ltd., Malvern, UK). The scattering movement and the size distribution of liposomes were also determined with a complementary method, ZetaView^®^ Nanoparticle Tracking Analysis (NTA, Particle Metrix GmbH, Inning am Ammersee, Germany).

#### 4.4.2. Microscopy Imaging

The morphology and internal structure of BBR-loaded liposomes were investigated using two microscopic techniques: Cryo-EM (FEI Talos Arctica, Thermo Fisher Scientific Inc., Waltham, MA, USA) and fluorescence microscopy (Leica TCS SP8, Leica Microsystems GmbH, Wetzlar, Germany).

For Cryo-EM, the vitrified samples were prepared from 3 μL aliquots with Leica EMGP vitrification device (Leica Microsystems GmbH, Wetzlar, Germany) on freshly glow-discharged Quantifoil R1.2/1.3 grids. The samples were observed in a FEI Talos Arctica microscope operated at 200 kV. The image stacks were recorded at a nominal magnification of 150,000× (calibrated pixel size 0.97 Å/pxl) with a FEI Falcon 3 camera, and subsequently, motion corrected with software package Scipion.

For optical microscopy, the liposomes samples were placed onto glass-bottom dishes and imaged using an inverted confocal microscope. A plant-origin BBR exhibits fluorescence when excited at the wavelength of 350 nm and shows an emission spectrum ranging from 470 to 670 nm [44]. We used a 488 nm argon laser for excitation and detected the signal at 500–570 nm range. Another method to visualize BBR distribution in liposomes was based on two-photon excited fluorescence (TPEF). In the case of TPEF, the source (picoEmerald, APE, Berlin, Germany) was tuned to wavelengths between 800 nm and 820 nm and the emitted BBR fluorescence was detected at around the 400–500 nm range.

#### 4.4.3. Determination of Entrapment Efficiency (EE)

For determining the free drug, 1.0 mL of BBR-loaded liposomes suspension was filled in the Ultra centrifugal filter type (MW 30,000 dalton, Merck KGaA, Darmstadt, Germany) and subsequently centrifuged at 4000 rpm for 30 min. The centrifugal fluid was collected and placed in a 10 mL volumetric flask. Absolute ethanol was filled up to the pre-set mark and the volumetric flask was shaken to mix the solution. The solution was further diluted with 96.5% (*w/v*) ethanol to obtain a suitable concentration. The absorbance of BBR was measured with a UV-Vis spectrophotometer (UV-1800, Shimadzu, Kyoto, Japan) at a wavelength of 350 nm (the maximum absorption wavelength of BBR in 96.5% (*w/v*) ethanol). The concentration of free BBR (*C_free_*) in centrifugal fluid was calculated using a following standard curve: y = 52.988 x −0.0026 (R^2^ = 0.9899; *n* = 6). The standard curve was constructed from a series of standard solutions with the BBR concentrations ranging from 4 µg/mL to 12 µg/mL.

For determining the total BBR concentration (*C_total_*), another 1.0 mL of BBR-loaded liposomes suspension was placed in a 10 mL volumetric flask. The following steps were the same as in the previous experimental protocol. Absolute ethanol was filled up to the pre-set mark. The volumetric flask was shaken for breaking up the liposomes and diluting the solution. Then, the solution was further diluted with ethanol 96.5% (*w/v*) to obtain a suitable concentration for UV-Vis spectroscopy. The total concentration (*C_total_*) was calculated. The blank solutions were prepared by the same protocol as described previously for determining *C_free drug_* and *C_total_* but using the empty liposome suspensions. The *EE* was determined using the following equation, Equation (2) [16]:

(2) $$\mathrm{EE}\;(\%)=\frac{\left(C_{\mathrm{total}}-C_{\mathrm{free}}\right)\;\times \;100\;}{C_{\mathrm{total}}}$$

#### 4.4.4. Drug Release In Vitro

The in vitro dissolution tests of BBR-loaded liposomes were carried out using a dialysis method at 37 °C. The dialysis tubing cellulose membrane (MW cut-off = 14,000 Da, average flat width 25 mm; Sigma-Aldrich, Corp., St Louis, MO, USA) was pre-soaked in release media for 12 h in order to wet the membrane. Three different media were used in the experiments: a diluted 0.1N hydrochloric acid (HCl) solution (pH 1.2), phosphate buffer saline (pH 4.5) and phosphate buffer saline (pH 6.8). Briefly, 6.0 mL of BBR liposomes suspension (the average concentration of BBR in the liposome suspension 0.5 mg/mL) and 4.0 mL of release medium were placed in dialysis bags, which were then sealed. The dialysis bags were mounted on the paddles of a dissolution tester (Erweka GmbH, Langen, Germany) before immersion into the release media. The pure BBR solution (0.5 mg/mL) was prepared in the same way. Each vessel of the dissolution apparatus was filled with 300 mL of the medium. The temperature of the dissolution media was kept at 37 °C and the rotating rate of the paddles was set at 50 rpm. The samples (5 mL) were withdrawn at regular intervals after 0.25, 0.5, 1, 2, 3, 4, 6, 8, 10, 12 and 24 h and immediately replaced with an equal volume of fresh medium after each sampling. The content of BBR in the release media was determined by means of spectrophotometry (U-1800, Hitachi High-Tech Corp., Tokyo, Japan). The release rate (RR) of BBR in the dialysate was calculated using the following Equation (3) [17]:

(3) $$\mathrm{RR}\;\left(\%\right)=\frac{C_{n}\times \;V_{m}\;+\Sigma_{i=1}^{i=n-1}C_{n-1}\;\times \;V\;}{W_{\mathrm{total}}\;}\times \;100\%$$

where *C_n_* is the concentration of BBR in the release medium at the time point when the sample number n was withdrawn, *V_m_* is the volume of release medium (300 mL), V is the volume of the release medium withdrawn at each sampling point (5 mL), n is the number of samples removed from the release medium, *C_n_*−_1_ is the concentration of BBR in the release medium at the time point when the sample (*n*−1) was removed, *W_total_* is the total amount of BBR in the BBR liposomes prior to dialysis. The drug release experiments were conducted in three media and repeated three times with each liposome formulation tested.

#### 4.4.5. Physical Solid-State Properties

The BBR containing liposomes in an aqueous suspension were dried at 40 °C and −0.09 mPa for 48 h in a vacuum oven (Daihan Labtech Co., Ltd., Gyeonggi-Do, South-Korea) to obtain a dried powder. The liposomes in powder form, the excipients (HSPC, DSPG, SDC) and the physical mixtures of BBR and the excipients were uniformly spread on a glass substrate and placed in an X-ray diffractometer (Equinox 5000, Thermo Fisher Scientific, Illkirch-Graffenstaden, France) with Cu-Kα1 radiation, monochromatized by a secondary flat graphite crystal. The scanning angle ranged from 0 to 120° 2*θ*, steps were of 0.015° 2*θ* and the counting time was of 100 s/step.

### 4.5. Statistical Analysis

A paired sample T-test was used for statistical analysis. The difference between the EE of the two methods (ethanol-injection and thin-film hydration) was tested at significance level *p* < 0.05.

## 5. Conclusions

Ethanol-injection and thin-film hydration are feasible methods for generating drug-loaded liposomes intended for the oral administration of poorly water-soluble BBR. Liposomes with a uniform size and relatively high active load can be fabricated. The size, structure, PDI and EE of the liposomes are dependent on the fabrication method and lipid molar ratio (HSPC:DSPG) used. The ethanol-injection method generates smaller and more uniform-sized BBR-loaded liposomes than the thin-film hydration method. The EE of such liposomes, however, is not as high as with the corresponding liposomes generated by the thin-film hydration method. The internal structure of the BBR-loaded liposomes generated by the ethanol-injection method is SUV and LUV, while the corresponding liposomes fabricated by the thin-film hydration method are UV-, MLV- and MVV-type liposomes. BBR accumulates in the bilayer phospholipid membrane of the liposomes, which can be verified by means of confocal fluorescence microscopy. The BBR-loaded liposomes introduced in our study present pH-dependent extended drug release behavior in vitro. More research is needed to verify whether this drug release pattern could advance the oral bioavailability of BBR.

## Figures and Tables

**Table 1:** Comparison of the size and polydispersity index (PDI) of berberine (BBR)-loaded liposomes fabricated by ethanol-injection (FJ) and thin-film hydration (FH) methods (after extruding 60 times through a polycarbonate membrane with a pore size of 0.4 μm) and analyzed with the Zetasizer Nano ZSP system (*n* = 3; mean ± SD).

Exp.	Formulation Codes	Ethanol-Injection		Thin-Film Hydration	
		Mean Size (d.nm)	PDI	Mean Size (d.nm)	PDI
1	FJ4, FH4	133.6 ± 1.1	0.209 ± 0.008	248.7 ± 3.3	0.272 ± 0.053
2	FJ21, FH21	117.3 ± 1.3	0.186 ± 0.004	236.0 ± 2.0	0.248 ± 0.009
3	FJ17	82.3 ± 1.3	0.113 ± 0.004	–	–
4	FJ13, FH13	149.1 ± 0.8	0.220 ± 0.008	182.8 ± 2.6	0.297 ± 0.022
5	FJ12, FH12	120.4 ± 1.1	0.243 ± 0.001	292.0 ± 3.9	0.181 ± 0.033
6	FH11	–	–	209.0 ± 1.1	0.317 ± 0.037
7	FJ14, FH14	243.6 ± 4.1	0.195 ± 0.012	208.2 ± 3.2	0.312 ± 0.031
8	FJ16, FH16	91.2 ± 2.0	0.232 ± 0.015	448.5 ± 6.6	0.276 ± 0.016
9	FJ18, FH18	50.9 ± 1.2	0.259 ± 0.009	239.2 ± 2.4	0.243 ± 0.009
10	FJ19, FH19	146.5 ± 2.7	0.186 ± 0.007	111.5 ± 3.7	0.285 ± 0.011

**Table 2:** The compositions of the berberine-loaded liposomes. Key: berberine (BBR), sodium deoxycholate (SDC), alpha-tocopherol (α-TP), phosphatidyl choline (HSPC) and distearoyl phosphatidyl glycerol (DSPG).

Exp.	Formulation Code	Molar Ratio (BBR:Lipid:SDC α-TP)	Lipid Molar Ratio (HSPC:DSPG)
1	F04	9:9:2:0	7:3
2	F21	9:9:0:3	7:3
3	F17	8:9:2:2	7:3
4	F13	8:9:2:2	6:4
5	F12	8:9:2:0	7:3
6	F11	8:9:2:0	4:6
7	F14	6:9:2:0	6:4
8	F16	6:9:2:0	4:6
9	F18	6:9:2:0	7:3
10	F19	8:9:2:2	4:6

## Data Availability

Not applicable.

## Supplementary Materials

The following are available online. Video S1: Scattering movement of liposomes FJ17; Video S2: The 3D rotation fluorescent microscopy image of liposomes FH12.

## Sample Availability

Samples of the compound berberine, BBR (Sichuan Weikeqi Biological Technology Co., Ltd., Chengdu, China), are available from the authors.
